# Gender Reassignment and the Role of the Laboratory in Monitoring Gender-Affirming Hormone Therapy

**DOI:** 10.3390/jcm13175134

**Published:** 2024-08-29

**Authors:** Indra Ramasamy

**Affiliations:** Conquest Hospital, Hastings TN37 7RD, UK; indrar@ozemail.com.au

**Keywords:** gender incongruence, gender-affirming hormone treatment, laboratory tests in transgender individuals

## Abstract

Transgender people experience distress due to gender incongruence (i.e., a discrepancy between their gender identity and sex assigned at birth). Gender-affirming hormone treatment (GAHT) is a part of gender reassignment treatment. The therapeutic goals of the treatment are to develop the physical characteristics of the affirmed gender as far as possible. Guidelines have been developed for GAHT, which recommend dosage as well as different formulations of oestrogen and testosterone for treatment. Questions arise about the metabolic side effects of hormone treatment. Establishing reference ranges for common analytes in transgender individuals remains a task for laboratory medicine. It has been suggested once GAHT is commenced, the reference ranges for affirmed gender are reported for red blood cells, haemoglobin and haematocrit. For transgender assigned-female-at-birth (AFAB) people, testosterone concentrations are recommended to be within the reference interval established for cisgender men and for transgender assigned-male-at-birth (AMAB) people, estradiol concentrations are within the reference range for cisgender women. Sex-specific reference ranges are available for certain laboratory tests, and these may be organ (e.g., heart)-specific. Transgender-specific reference ranges may be a requirement for such tests. Laboratories may need to make decisions on how to report other tests in the transgender population, e.g., eGFR. Interpretation of further tests (e.g., reproductive hormones) can be individualized depending on clinical information. Electronic medical record systems require fields for gender identity/biological sex at birth so that laboratory results can be flagged appropriately. In this review, we aim to summarise the current position of the role of the laboratory in the clinical care of the transgender individual. Prior to the review, we will summarise the genetics of sex determination, the aetiology of gender incongruence, and the recommendations for GAHT and monitoring for the transgender population.

## 1. Introduction

Gender incongruency (GI) occurs if the gender identity expressed by the individual and the biological sex of the individual are not consistent with each other. GI may be associated with distress, unease, depression, and low quality of life, which in most cases improve during gender-affirming hormonal treatment (GAHT). In many countries, GAHT is prescribed following an initial assessment by multidisciplinary teams. The teams offer initial psychological support prior to prescribing GAHT [1]. Recent guidelines describe the initial assessment and treatment of GI adolescents and adult individuals. In the scientific literature, transgender people are usually categorised as transgender assigned-male-at-birth (AMAB) people and transgender assigned-female-at-birth (AFAB) people. Guidelines further suggest standard monitoring plans for transgender AFAB and AMAB people treated with GAHT [2,3,4,5]. GAHT can affect laboratory-based diagnostic measurements. Results can be difficult to interpret for physicians due to the absence of published reference intervals.

In this review, we summarise the genetics of sex development, the possible origins of GI, GI treatment, and suggested monitoring following GAHT. This review has a special focus on laboratory interpretation of common tests following GAHT.

## 2. Method

This narrative review covered several topics, and terms (e.g., gender identity, gender-affirming hormone treatment) were used to identify reviews or articles in PubMed, Medline, Google Scholar, and Web of Science over the past 10 years. From each article/review we extracted further references for studies included in this article.

## 3. Gender Development

Gender incongruence refers to the discordance between biological sex and gender identity, i.e., children or adults who do not identify with their biological sex. Multiple terms have been used for gender incongruence in the scientific literature, such as transsexualism, transgenderism, gender identity disorder, and gender dysphoria. Minor differences in nuances occur in each term, and the term GI is used as a common term in this review [6]. Prior to examining gender incongruency, this review will first summarise specific genetic signals that ensure sex development in humans. Sex development includes three distinct sequential stages: development of the bipotential gonad; sex determination, gonadal differentiation into testes and ovaries; and sex differentiation, the development of external and internal genitalia, or phenotypic sex. In humans, the bipotential gonads originate at 5 weeks of gestation from the gonadal ridge. The primordial germ cells, precursors to sperm and eggs, actively migrate across the embryo to reach the bipotential gonads [7,8]. Multiple essential genes are involved in bipotential gonadal development. Many of the genes were first reported in animal models. A number of genes (including transcription factors, PBX1, EMX2, and CBX2) [7,9] have been implicated in early gonadal development. Knowledge of factors involved in sex development came from animal models or from case studies in which the genetic or gonadal sex does not equal phenotypical sex, termed disorders of sex development (DSD). As an overall generalisation, factors influencing sex determination are transcriptional regulators, and factors responsible for sex differentiation are secreted hormones and their receptors.

In XY embryos, increasing levels of SF1 (an orphan nuclear receptor) and Wilms Tumour Suppression (WT1) gene, the gonadal development genes, activate the SRY gene expression on the Y chromosome. The SRY gene, the Y chromosomal testis determining gene, initiates the differentiation of Sertoli cells, which develop Leydig cells and germ cells. SRY regulates SOX9 gene expression, which promotes cell differentiation. Several other genes that participate in testicular development are described by Reyes et al. [7]. In males, sex differentiation begins at 7 weeks gestation. Sertoli cells express factors that differentiate and develop Sertoli cells, Leydig cells, and germ cells [10]. Anti-Müllerian hormone (AMH) is one of the earliest cell-specific proteins formed by the Sertoli cells. During male fetal development, AMH provokes the regression of the Müllerian duct, the rudiments of the fallopian tubes, the uterus, and the upper part of the vagina [11]. Human chorionic gonadotrophins (hCG) produced by the placenta in the first trimester of pregnancy and luteinizing hormone (LH) secreted by the pituitary in mid-gestation stimulate Leydig cells to induce testosterone production. Leydig cells respond to hCG/LH, which binds to the LH/hCG receptor and enhances the activity of enzymes that increase testosterone production. Testosterone binds to the androgen receptor in the Wolffian duct to form the male gonaducts. Testosterone is formed into dihydrotestosterone (DHT) by the enzyme 5a-reductase. DHT binds to the androgen receptor with higher affinity than testosterone and drives the differentiation of male external genitalia [12]. The testes are initially located close to the kidneys and, during development, migrate to the lower abdomen and then through the inguinal canal to the scrotum in a hormone-independent process [7].

**Figure 1:**
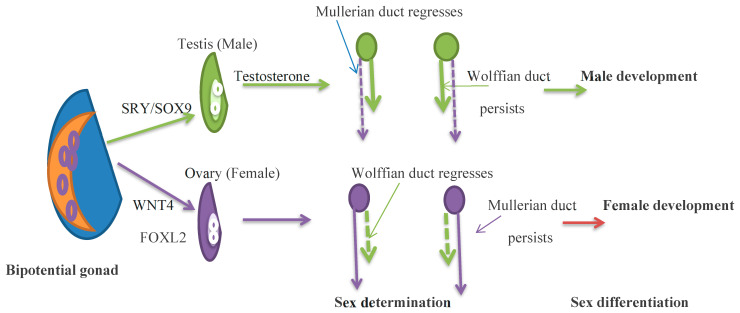
Germ cells migrate to the gonadal precursor to form the bipotential gonad by 5 weeks of gestation. At 6–8 weeks of gestation, SRY genes, expressed in somatic cells, drive testis determination, and in the absence of the SRY gene, FOXL2 and WNT4 drive ovarian determination. In the XY embryo, testosterone develops the Wolffian duct, and the anti-Müllerian hormone regresses the Müllerian duct. In the XX embryo, the Müllerian duct persists and the Woffian duct regresses. By 12 weeks of gestation, external genitalia are observed. Green shows the development of the male gonad, and purple shows the development of the female gonad.

In the absence of the SRY gene, the XX embryo expresses multiple pro-ovarian genes, which include FOXL2, WNT4, and RSPO1. These factors support ovarian differentiation and suppress testis development. FOXL2 maintains granulosa cell differentiation and supports folliculogenesis during development and adulthood. In the female, in the absence of testicular hormones, effective genetic regulation causes Wolffian duct regression and the development of fetal internal genitalia from Müllerian ducts as well as the growth of female external genitalia. The absence of AMH allows the Müllerian ducts to persist and form the fallopian tubes, uterus, and upper third of the vagina. AMH production begins after Müllerian duct differentiation by the counterpart of Sertoli cells, the granulosa cells. AMH is a folliculogenesis regulator and a biomarker of the primordial follicle reserve (Figure 1) [7].

One of the first hormonal changes in puberty is the pulsatile release of GnRH, which stimulates the release of LH and follicle-stimulating hormone (FSH). LH acts on the theca cells of the ovary to increase estrogen production and the Leydig cells of the testis to increase testosterone. In the female, FSH works on the ovarian follicle to convert oestrogen precursors to oestrogen and within the male Sertoli cells of the testes to form sperm. This results in the formation of the adult male genitalia and in the female development of the breasts. The adrenal gland contributes to the formation of secondary sexual characteristics, particularly the development of pubic and axillary hair, termed pubarche. Tanner staging is an objective classification system that documents the development of secondary sex characteristics in children during puberty [13] (Table 1).

### Genesis of Gender Incongruence

Despite the increase in gender health research, little is known about the timing of gender identity crystallization and the factors that contribute to the development of a gender identity that is not consistent with the sex determined by chromosomal or biological sex. Factors associated with gender incongruence and the scientific evidence in the literature require further exploration. With the advances in neuroimaging, one suggestion is that differences between male and female human brains may be trivial and population-specific [14]. Ruigrok et al. [15] report on regional brain differences between males and females, which are due to biological and environmental influences. Sex differences in gene expression have been reported in the brain, and sex hormones can further influence brain morphology [16]. Despite decades of research, sex differences in brain function are only partly understood. Gender expression is further likely to be a complex interplay of cultural and environmental factors (psychosocial factors) [17]. Gender identity may be an expression of a complex interplay between biological and environmental pressures.

Publications suggest that the biological origin of transgender identity is based on atypical sexual differentiation of the brain (transgender-specific brain phenotype) [18] or the hormone milieu during intrauterine development. Twin studies suggest a heritable component in transgender identity [19]. Foreman et al. [20] found a significant association between gender incongruence and SRD5A2 and STS alleles, as well as ERα and SULT2A1 genotypes in their cohort of transgender AMAB people. The authors suggest a polygenic basis for the transgender AMAB phenotype. Fernandez et al. [21] suggest that specific genotypic combinations of oestrogen and androgen receptors are associated with the transgender population.

Currently, there is no objective criterion for gender incongruence, and the literature suggests that GI has multiple aetiologies. Understanding the aetiology of GI would help clinicians decide which type of intervention would help in each individual case.

## 4. Guidelines for GAHT

### 4.1. Adolescent GI

One suggestion is that most children develop an ability to label their own and others’ genders between 18 and 24 months, and for the majority of adolescents, gender identity agrees with the assigned gender [22]. One review suggests that 1.2–2.7% of children and adolescents and 0.3–0.5% of adults identify as transgender [23]. There is ongoing debate about how children with GI should be treated and how their rights should be respected. The unease with which GI presents in prepubescent children varies; it is often transient and does not continue once puberty begins. Other children with GI exhibit a constant desire to be of the other gender and to match the physical and sexual characteristics of the desired gender. In addition, detransition—or reversing gender transition—can occur in adolescents and young adults [24].

Treatment follows an all-inclusive multidisciplinary clinical and psychosocial assessment of the GI individual, which includes both counselling and support. Treatment includes the following: (1) suppression of puberty by Gonadotropin-Releasing Hormone analogues (GnRHa, or puberty blockers); (2) administration of gender-affirming cross-sex hormones; and (3) gender-affirming surgery [25].

The European Academy of Pediatrics (EAP) states that ‘the child’s best interests are the primary consideration.’ The provision of puberty blockers and gender-affirming therapy in children under 18 years old is under critical review [24]. There has been controversy around the use of GnRHa to block puberty in peri-pubescent children. GnRHa treatment is reversible. GnRHa treatment gives the adolescent time to reconsider while reducing the development of secondary sexual characteristics. Delay may cause psychological and physical harm, though others have stated that there is no evidence for the latter. Studies suggest that the majority who started GnRHa treatment continued with gender-affirming treatment [26,27]. More studies are needed to describe the effect of transgender hormonal treatment on the skeleton and on brain development during adolescence. For the transgender population, while puberty suppression alone does not affect fertility outcomes, the addition of cross steroids does interfere with reproductive potential [28]. A recent review (Cass review) [29] of the Bell vs Tavistock High Court Case (UK) states that gender-affirming care is not backed by strong evidence on the natural history of GI and the efficacy of treatment alternatives. The following two critical questions need to be answered for pre-pubertal GI patients: (i) is the transition pathway beneficial for the individual? and (ii) is the pathway consistent with the ‘do no harm’ principle [29]? A suggestion is that the decision to treat adolescent GI made by a healthcare professional should be based on individual needs and scientific evidence [30]. It is expected that as experience with puberty suppression in transgender AFAB and AMAB children increases, there will be progress in understanding the best ways to provide endocrine care to transgender GI children, although further studies are needed to investigate adverse events.

### 4.2. Adult GI

In adult GI, the goals of hormone treatment are to reduce endogenous sex hormone levels and to replace hormones with sex hormone levels consistent with the individual’s gender identity. In transgender AFAB people, several androgen preparations have been used to achieve physiological levels consistent with the individual’s gender identity. Treatment for transgender AMAB people involves either oral or transdermal 17β-estradiol. Other adjunctive therapy is used to reduce endogenous testosterone levels. Progestins with anti-androgen activity, GnRH agonists, and spironolactone are some of the medications available [2]. A summary of recommendations from three different worldwide organisations’ published guidelines is provided in Table 2 [2,3,4,5]. It has been suggested that future guidelines might address the holistic healthcare of transpeople by increasing the evidence base, upgrading the quality of clinical practice guidelines, and increasing the number of health topics considered for the transgender population [31]. Individual goals for non-binary transgender AFAB and AMAB people can be complex, and individualised treatment is suggested. Ideal patient-centered outcomes for GAHT need to be defined for the non-binary population. However, adjusting hormonal treatment to attain some characteristics and not others can be a challenge [5]. Further research is needed to guide individualised hormonal treatment and clinical care in transgender AFAB/AMAB people.

## 5. Laboratory Tests in Transgender AFAB/AMAB Individuals

Laboratory tests are affected by gender-affirming feminising or masculinising therapy or puberty-suppressing treatment. Laboratory tests are recommended by expert opinion or clinical practice guidelines [2,3,4,5]. Some tests are baseline tests prior to treatment, and others are used for treatment monitoring (Table 3 and Table 4). In addition, transgender individuals may receive laboratory tests for other clinical indications. Laboratory tests likely affected by gender-affirming treatment are those that have sex-specific reference intervals, which may, additionally, be target-organ-based.

Guidelines concur in that for transgender AMAB people, the suggested estradiol levels are aimed at the adult reference range with suppressed testosterone, and for transgender AFAB people, the suggested testosterone levels are within the adult reference range. Blood tests measure serum estradiol to monitor treatment but cannot monitor synthetic oestrogen use, and clinicians use serum estradiol to monitor treatment. Monitoring frequencies are similar in guidelines developed by WPATH and AusPATH [3,5]. Recommended ranges are used as a guide, and how the patient responds to treatment and associated risk factors that are present may guide GAHT (AusPATH). Other tests suggested are full blood count, electrolytes, renal and liver function tests, glucose, and lipids (AusPATH). Monitoring hormone concentrations as well as physiological changes can be used to optimise gender-affirming therapies and minimise adverse events.

### 5.1. Red Blood Cell Indices

Several prospective studies have investigated the effect of taking gender-affirming hormones on some analytes. Studies are not always powered to analyse subgroups with differences in medication and dosage and route of hormones or analyte measurement carried out on different analyser platforms. Humble et al. [32] report on transgender people treated with hormone therapy for at least 6 months. In transgender individuals receiving masculinising hormones, when compared to baseline levels, creatinine, red blood cells (RBC), haematocrit, haemoglobin, and testosterone were increased, similar to previous studies, and HDL decreased. In transgender individuals receiving feminising hormones, RBC, haematocrit, haemoglobin, testosterone, and creatinine levels were decreased when compared to baseline levels. SoRelle et al.’s [33] study of transgender individuals on hormone therapy for more than 6 months reported similar changes in RBC, haematocrit, haemoglobin and creatinine. In a small study of transgender AFAB people treated with testosterone, the increase in creatinine and RBC indices was stable for 5 years. In transgender AFAB off GAHT, haemoglobin decreased to the female range in 17 weeks. The study suggests that for RBC indices, reference ranges for a person’s affirmed gender apply once on stable GAHT [34] (Table 5). Other studies have reported similar findings in RBC indices in transgender adolescents following GAHT. The authors did not report other significant laboratory abnormalities in transgender adolescents receiving GAHT [35].

Women and men have different levels of haemoglobin, which is probably the effect of oestrogens and androgens on erythropoiesis [36]. Greene et al. [37] reviewed haematology reference ranges for healthy transgender AMAB/AFAB individuals. The oestrogen-treated cohort had values similar to those of cisgender women, and the testosterone-treated cohort had values similar to those of cisgender men.

### 5.2. Renal Function

It has been suggested that changes in muscle mass in transgender individuals can contribute to changes in serum creatinine. This raises questions about the calculation of eGFR, which uses sex-based calculations and has implications for the estimation of kidney function. This has consequences for the administration of agents (e.g., intravenous contrast agents) that may impact kidney function, kidney transplant eligibility, or renal failure class allocation. One alternative is to use a more direct measure of GFR estimation such as 24 h urine creatinine clearance [38].

### 5.3. Liver Enzymes

Studies on the effect of GAHT on transaminases are conflicting. At least two studies report that changes in transaminases are not likely to be of clinical significance [33,39]. A further study suggests that the interpretation of transaminase and alkaline phosphatase levels are affected by gender-affirming testosterone therapy and recommends the use of affirmed gender reference intervals [40].

### 5.4. Lipids

Mixed results across multiple studies have been reported for total cholesterol, triglycerides (TG), LDL, and HDL in transgender individuals receiving GAHT [41,42,43,44].

### 5.5. Cardiac Biomarkers

In a further cross-sectional study, similar to healthy cisgender people, transgender AFAB people have higher concentrations of high-sensitivity troponin and lower concentrations of N-terminal pro-brain natriuretic peptide compared with transgender AMAB people [45]. In a small study with a single cut-off value of high-sensitivity troponin I and gender-specific reference ranges, 1.1% of patients would have been reclassified as acute myocardial infarction if the threshold value was based on the gender assigned at birth instead of their affirmed gender identity [46].

### 5.6. Reproductive Hormones

The distribution of endocrine results for estradiol, SHBG, prolactin, AMH, FSH, LH and testosterone for healthy transgender AMAB people differed from that for cisgender men and cisgender women. Treatment with spironolactone had a significant effect on the distribution levels of these hormones [47]. For transgender AFAB people, the distributions of testosterone and SHBG are similar to those of cisgender men. The distribution of results for estradiol, FSH, LH, progesterone, and prolactin differed from those for cisgender men and women, and AMH and dehydroepiandrosterone (DHEAS) differed from cisgender women [48]. It is suggested that reproductive hormone results should be interpreted in a manner specific to the transgender population.

### 5.7. Ferritin

Serum ferritin levels are influenced by dietary intake of iron, alcohol intake, chronic liver disease, and inflammatory disorders. Reference ranges for serum ferritin vary according to age and sex. Ferritin reference ranges are lower in premenopausal women compared to postmenopausal women. Female reference ranges are typically lower than those of men. There are no studies on the effect of GAHT on serum ferritin levels [49]. When a diagnosis of iron overload is suspected, and secondary causes are excluded, genetic studies for primary haemochromatosis may be indicated.

### 5.8. Prostate Specific Antigen

In transgender AMAB people, it is rare for the original prostate to be removed during orchiectomy. There is a risk of prostate cancer as long as the prostate remains in situ. Overall, transgender AMAB people showed a lower risk of prostate cancer compared to cisgender women [50], though it may not be as uncommon as previously supposed [51]. Reports suggest a more aggressive presentation than in cisgender men, with metastatic disease on presentation [52]. Little is known about prostate cancer screening in the transgender AMAB population. Future research avenues are the threshold values for prostate-specific antigen (PSA), which should be considered elevated for those on GAHT.

**Table 5:** Impact of GAHT on laboratory tests.

Laboratory Tests			Comments	Reference
	Estradiol treatment	Testosterone treatment	Estradiol GAHT shifts haemoglobin, haematocrit to lower values in line with cisgender women’s reference intervals. Testosterone GAHTshifts reference intervals to higher levels in line with cisgender men’s reference intervals	[32]
RBC	Decrease	Increase		
Hemoglobin	Decrease	Increase		
Hematocrit	Decrease	Increase		
Creatinine	Decrease	Increase	The most reno protective calculated GFR either male/female is suggested; 24h creatinine clearance if indicated	[38]
High sensitivity troponin I			Report a reference range that would allow critical results to be appropriately followed; an approach of least harm to the patient is suggested	[45]
Ferritin			Laboratories use dual reference ranges for cisgender individuals. Interpretation is based on clinical presentation (e.g., pregnancy) in combination with full blood count, liver function test, and markers of inflammation, e.g., CRP. Iron overload: If secondary causes excluded, investigation for primary haemochromatosis gene may be indicated	[49]
Reproductive hormones	Testosterone, Estradiol		Following stabilisation of treatment with gender-affirming hormones, guidelines suggest treatment goals are physiological levels of the affirmed gender identity cisgender adults. The time of measurement of the hormone is dependent on the method of administration as well as formulation of the GAHT	[2]
Reproductive hormones			LH, FSH, AMH, and DHEAS are variable in a transgender population and are interpreted with clinical information	[47,48]
PSA			Data for reference ranges in transgender AMAB people and from screening for prostatic cancer is not available	[52]
Renal function/liver function/lipid profile			Guidelines suggest monitoring of liver function/renal function and lipids during GAHT treatment. Sex-specific reference ranges are not ordinarily stated for the measurements	[44]

### 5.9. Laboratory Test Reference Intervals for Transgender Population

The reference interval for clinical laboratory tests is a requirement. They are necessary for the correct interpretation of tests and direct the care of the intended population. GAHT is medically indicated in transgender patients. To help clinically manage transgender patients, reference intervals have to take the effect of treatment on laboratory results into account. A summary of recent advances is given in Table 5. Interpretation may still need to be individualized, especially for individuals on a nonstandard treatment regimen of GAHT, during the initial treatment prior to stabilization of therapy, or with co-existing medical conditions.

One principle [53,54] for the selection of different ranges for patients who have started therapy is the organs and physiological hormones influenced by GAHT. Individuals assigned as male at birth have larger organs, such as heart and muscle, following puberty. The reference range for troponin and creatinine may differ. GAHT can influence erythropoiesis, lipid parameters, and reproductive hormones.

## 6. Electronic Medical Record Systems (EMR)

From the laboratory perspective, the appropriate capture of gender information can have several implications, from test ordering to information gathering on the variation of analytes (i.e., to set up analyte reference ranges). The inclusion of gender identity in the EMR and, if the individual chooses to disclose this information, gender identity at birth can be relevant to individual treatment decisions and help in individual care [55]. In the USA, electronic medical records and laboratory information systems have the capacity to capture gender identity information. However, the introduction of the system into medical records can be challenging [56]. As a result of current limitations in the EMR systems, interactions with laboratory services can increase distress to transgender AFAB/AMAB individuals and affect their mental health.

## 7. GAHT and Other Laboratory Markers

### 7.1. Risk of Venous Thromboembolism in AMAB People

In cisgender females, treatment with oral contraceptives increased the risk of venous thromboembolism 2–4 fold, whereas the transdermal oestrogen formulation used for hormone replacement treatment does not appear to be associated with a significant venous thromboembolism risk. In a meta-analysis, Totaro et al. [57] suggest that the overall risk of venous thromboembolism in transgender AMAB people undergoing gender affirmation treatment was 2% but was negligible in those <37.5 years. Other studies confirm that the risk of venous thromboembolism during cross-hormone treatment is rare [58], though the risk may be modified by type, dose, route of oestrogen, duration of treatment, increasing age, high BMI, and smoking [59]. Prothrombotic variants, Factor V Leiden, prothrombin G2010A mutation, Protein S deficiency, Protein C deficiency, and antithrombin deficiency can increase the risk of hormone treatment. Previous venous thromboembolism and family history of genetic thrombophilia are considered reasons for thrombophilia screening prior to hormone treatment [2].

### 7.2. Hyperprolactinemia

Studies report hyperprolactinemia among transgender AMAB people taking both oestrogens and an antiandrogen [60]. The authors found too few cases of prolactinoma in transgender AMAB people on gender-affirming treatment to draw a conclusion. A threshold value for the definition of hyperprolactinemia in transgender AMAB people needs to be established.

### 7.3. Other Sex Hormone Dependent Tumours

There is little evidence about the effect of GAHT on the development of hormone-dependent cancer among transgender individuals. The evidence for most aspects of breast cancer in transgender AFAB people is inadequate [61,62]. However, one suggestion is that transgender AFAB people carrying a breast cancer mutation should be investigated further. Specific guidelines for breast cancer screening, intended for transgender AFAB people prior to mastectomy, mimic guidelines for cisgender women [61].

### 7.4. Bone Mineral Density

Sex steroids contribute to bone growth and peak bone mass accumulation during puberty and in adults contribute to the maintenance of bone structure. In transgender AFAB/AMAB adolescents, blocking puberty with gonadotropin-releasing hormone analogues decreases bone mineral density (BMD). Commencement of GAHT at least partially reverses the bone loss associated with pubertal suppression [63]. A review of studies suggests that GAHT in transgender AFAB people does not compromise bone microarchitecture. A summary of several systematic reviews indicates that reports on the effect of GAHT on the bone health of transgender AMAB are inconsistent [64,65]. Some data support the statement that pharmacological oestrogen can increase bone mineral density in transgender AMAB people [66].

The Endocrine Society clinical practice guidelines for gender-incongruent individuals suggest checking bone density in patients who have risk factors for osteoporosis [2], such as hyperparathyroidism or steroid use.

It is not certain as to which database to use for the interpretation of BMD although it is possible to use both male and female databases for reference in the DXA report. The official position of the International Society for Clinical Densitometry (ISCD) is that transgender individuals should use the reference data of the gender conforming to the individual’s gender identity. If the referring provider or the individual requests, a set of male or female Z-scores can be provided to calculate the Z-score against male and female reference data, respectively [67,68]. Algorithms used to predict fracture risk, such as FRAX, use data derived from cisgender cohorts. These algorithms may not be able to correctly calculate fracture risk in the transgender population.

## 8. GAHT, Vascular Health and Cardiovascular Disease, and Impact of Aging in Transgender Adults

In a systematic review, van Leerdam et al. [69] suggest that GAHT reduces gender dysphoria and body dissatisfaction with a subsequent improvement in psychological well-being and quality of life, though they suggest further studies are indicated. Aggressive modification of cardiovascular risk factors, e.g., optimisation of diabetes, weight, and lipid profile, may be recommended in transgender patients under treatment with GAHT. Case studies suggest thrombotic risk assessment is indicated in at-risk patients [70]. The effects of GAHT on cardiovascular effects are difficult to assess due to the limited number of studies and contradictory outcomes [71]. There is a lack of research on treatment with GAHT during menopause and older age. Shared decision-making for treatment with GAHT in older age to minimise potential adverse effects has been suggested [72].

## 9. Conclusions

A cascade of complex genetic interactions leads to the formation of male and female phenotypes [73]. The disparity between the sex assigned at birth and the experienced gender or gender identity in GI individuals can cause distress. GI involves multiple aetiologies, and studies suggest the concept that genetic, endocrine, and neuroanatomic as well as a complex interplay of environmental and cultural factors, contribute to GI [2]. In some GI individuals, this distress is so great that they seek medical treatment to cause changes which match their gender identity. Guidelines suggest puberty suppression therapy for transgender AFAB/AMAB adolescents and testosterone and estradiol treatment for young adults who require transition treatment. Several guidelines suggest blood examinations and clinical evaluations should be performed at baseline and following GAHT treatment. A number of the recommended laboratory tests have been shown to be affected by GAHT. Laboratory tests impacted by GAHT are predominantly tests that have sex-specific reference intervals or are based on target organs affected by the biological sex of the individual.

## 10. Future Directions

A future informatics challenge is to use EMR systems to provide reference intervals and interpretative comments for laboratory tests ordered for transgender AFAB/AMAB individuals receiving GAHT. A study to create a comprehensive data set that can be used for a wide range of purposes and to address current controversies and improve care for GI individuals is a further task in this subject [74].

## Figures and Tables

**Table 1:** Tanner staging.

Tanner Stage	Pubic Hair (Male and Female)	Breast Development (Females)	Testicular Volume (Males)
1	No hair	No glandular breast tissue palpable	Testicular volume < 4 mL or long axis < 2.5 cm
2	Downy hair	Breast bud palpable under the areola (1st pubertal sign in females)	4–8 mL (or 2.5 to 3.3 cm long), 1st pubertal sign in males
3	Scant terminal hair	Breast tissue palpable outside areola; no areolar development	9–12 mL (or 3.4 to 4.0 cm long)
4	Terminal hair that fills the entire triangle overlying the pubic region	Areola elevated above the contour of the breast, forming a “double scoop” appearance	15–20 mL (or 4.1 to 4.5 cm long)
5	Terminal hair that extends beyond the inguinal crease onto the thigh	Areolar mound recedes into single breast contour with areolar hyperpigmentation, papillae development, and nipple protrusion	>20 mL (or >4.5 cm long)

**Table 2:** Summary of guidelines on GAHT of transgender AFAB/AMAB persons.

Guidelines	Society of Endocrinology	The World Professional Association for Transgender Health (WPATH)	Australian Professional Association for Trans Health (AusPATH)
Evaluation of prospective patients	Clinicians can add gender-affirming hormones after multidisciplinary team (MDT) team has confirmed the persistence of GI and sufficient mental capacity to give informed consent to treatment. The clinicians and mental health practitioners must be trained to diagnose GI.	Health care professionals have competencies in the assessment of transgender and gender diverse people wishing gender-related medical treatment and consider the role of social transition together with the individual. Liaise with professionals from different disciplines within the field of transgender health prior to gender-affirming treatment	
Treatment	(i) Recommend against treatment of pre-pubertal youth with gender affirming hormones (ii) Recommend treatment of adolescents (optimal time Tanner stage 2) with gonadotrophin-suppressing GnRH analogues (iii) In adults endogenous hormones are suppressed and administered sex steroids are maintained in the physiological range for the affirmed gender	(i) Unlike pubescent youth and adults, prepubescent gender diverse children are not eligible to access medical intervention. consider consultation, psychotherapy, or both, for a gender diverse child and family/caregivers (ii) Tanner stage 2 has to be reached prior to puberty suppression treatment (iii) Gender-affirming medical interventions (hormonal treatment or surgery) for non binary people in the absence of “social gender transition” is recommended	Unless there is agreement among the parents, the adolescent, and medical practitioner regarding competence, diagnosis, and treatment, a Family Court order is required for access to gender-affirming puberty blockers, hormone treatment, and surgery for adolescents under 18 years old.
Puberty Induction Regimen	Transgender AMAB people: Increasing doses of oral or transdermal 17β-estradiol, until adult dosage is reached. In postpubertal transgender AMAB people, the dose is increased more rapidly. Transgender AFAB people: Increasing doses of testosterone until adult values are reached. In postpubertal males, the dose is increased more rapidly. Adult maintenance dose is to mimic physiological adult levels.	In eligible youth who have reached the early stages of puberty, the aim is to delay further pubertal progression with GnRHas until an appropriate time when GAHT can be introduced. In these cases, pubertal suppression is considered medically necessary.	
Treatment of transgender AFAB/AMAB people	Transgender AFAB people: treatment with both parenteral and transdermal testosterone Transgender AMAB people: Oral, transdermal or parenteral oestrogen. Antiandrogens: spironolactone, cyproterone acetate, GnRH agonist. Estradiol and testosterone are maintained at premenopausal female levels. Gender-affirming hormones are maintained at normal adult ranges	Transgender AFAB people: Masculinising treatment, usually with testosterone. Transgender AMAB people: treatment is usually with oestrogen and androgen-lowering medication.	Transgender AFAB people: masculinising treatment is with different formulations of testosterone Transgender AMAB people: Feminising treatment includes oestrogen and androgen blockers. It is usual to start with low doses and titrate upwards.
Monitoring	Periodic monitoring of hormone levels, metabolic parameters, and assessment of prostate gland, gonads, and uterus as well as bone density	Hormone levels are measured during gender-affirming treatment to ensure endogenous sex steroids are lowered and administered sex steroids are maintained at levels appropriate for the treatment goals of transgender people according to the Tanner stage.	For masculinising treatment, total testosterone levels are maintained at the lower male reference range, and for feminising treatment, estradiol is aimed to be within the female reference range.
Reference	[2]	[3]	[4,5]

**Table 3:** Baseline and follow-up protocols during suppression of puberty.

	Clinical Chemistry Tests	Other Tests
	LH, FSH, E2/T, 25(OH)D	Anthropometry: height, weight, blood pressure, Tanner stages
Suggested Interval	6–12 months	3–6 months
		Bone density using DXA
Suggested Interval		1–2 years
Reference		[2]

DXA, dual-energy X-ray absorptiometry; E2, estradiol; FSH, follicle stimulating hormone; LH, luteinizing hormone; T, testosterone; 25(OH)D, 25 hydroxy vitamin D. AusPATH guidelines suggest in addition, full blood count, liver and renal function tests, human chorionic gonadotropin (hCG) (if indicated or requested) vitamin D (if clinically indicated) and ECG, fasting glucose, lipids, HbA1c if cardiovascular risk factors are present. During follow-up protocol following induction of puberty, the following additional clinical chemistry tests are suggested: In transgender AFAB people: haemoglobin/haematocrit, lipids, testosterone, 25OH(D). In transgender AMAB people: prolactin, estradiol, 25OH (D).

**Table 4:** Monitoring of transgender AFAB/AMAB individuals following gender-affirming treatment.

	Laboratory Tests	Other Tests
Transgender AFAB people	T	Monitor for virilization
Suggested Interval	3 monthly until levels within adult range	Every 3 months the first year and then one or two times per year
	Haematocrit or haemoglobin	Screening for osteoporosis, cervical screening (if cervical tissue present), breast cancer screening as recommended
Suggested Interval	3 monthly for first year then one/two times per year	
	Lipids at regular intervals	
Transgender AFAM people	Serum T and estradiol	Feminisation
Suggested Interval	Every 3 months	Every 3 months the first year and then one or two times per year
	If treated with spironolactone, electrolytes	Routine cancer screening and bone density
	Every 3 months the first year and then annually	
Reference		[2]
